# Genetic Diversity Among SARS-CoV2 Strains in South America may Impact Performance of Molecular Detection

**DOI:** 10.3390/pathogens9070580

**Published:** 2020-07-17

**Authors:** Juan David Ramírez, Marina Muñoz, Carolina Hernández, Carolina Flórez, Sergio Gomez, Angelica Rico, Lisseth Pardo, Esther C. Barros, Alberto E. Paniz-Mondolfi

**Affiliations:** 1 Grupo de Investigaciones Microbiológicas-UR (GIMUR), Departamento de Biología, Facultad de Ciencias Naturales, Universidad del Rosario, Bogotá 111211, Colombia; claudiamarina23@gmail.com (M.M.); dcahernandezc@gmail.com (C.H.); 2 Instituto Nacional de Salud, Bogotá 110911, Colombia; aflorez@ins.gov.co (C.F.); sgomezr@ins.gov.co (S.G.); arico@ins.gov.co (A.R.); lpardo@ins.gov.co (L.P.); ebarros@ins.gov.co (E.C.B.); 3 Icahn School of Medicine at Mount Sinai, New York, NY 10029, USA; Alberto.Paniz-mondolfi@mountsinai.org; 4 Instituto de Investigaciones Biomédicas IDB/Incubadora Venezolana de la Ciencia, Barquisimeto 3001, Venezuela

**Keywords:** SARS-CoV2, gene E, gene N, gene RdRp, PCR, molecular diagnosis, South America

## Abstract

Since its emergence in Wuhan (China) on December 2019, the Severe Acute Respiratory Syndrome Coronavirus 2 (SARS-CoV-2) has rapidly spread worldwide. After its arrival in South America in February 2020, the virus has expanded throughout the region, infecting over 900,000 individuals with approximately 41,000 reported deaths to date. In response to the rapidly growing number of cases, a number of different primer-probe sets have been developed. However, despite being highly specific, most of these primer-probe sets are known to exhibit variable sensitivity. Currently, there are more than 300 SARS-CoV2 whole genome sequences deposited in databases from Brazil, Chile, Ecuador, Colombia, Uruguay, Peru, and Argentina. To test how regional viral diversity may impact oligo binding sites and affect test performance, we reviewed all available primer-probe sets targeting the E, N, and RdRp genes against available South American SARS-CoV-2 genomes checking for nucleotide variations in annealing sites. Results from this in silico analysis showed no nucleotide variations on the E-gene target region, in contrast to the N and RdRp genes which showed massive nucleotide variations within oligo binding sites. In lines with previous data, our results suggest that the E-gene stands as the most conserved and reliable target when considering single-gene target testing for molecular diagnosis of SARS-CoV-2 in South America.

## 1. Introduction

The Coronaviridae comprises a large family of pathogenic viruses that are generally transmitted person-to-person through respiratory secretions or the fecal-oral route, but may also spread through zoonotic transmission [1]. Members of this family are generally spherical and slightly pleomorphic, ranging from 80 to 120 nm in diameter and covered by distinct projections, known as peplomers [2]. Coronaviruses (COVs) are enveloped positive-sense, single-stranded (ssRNA+) viruses [2] which are known to harbor the largest genomes amongst all known RNA viruses (27–32 kb) [2]. Overall, the genomic makeup of COVs embraces a variable number of small open-reading frames (ORFs) intercalated among different structural genes (ORF1ab, spike, envelope, membrane, and nucleocapsid) defining various lineages [2,3]. Current classification by the Coronaviridae Study Group (CSG) of the International Committee on Taxonomy of Viruses (ICTVs’) recognizes 39 species in 27 genera within the *Coronaviridae* family in the *Nidovirales* order which are known to infect a variety of vertebrates, including humans [4,5].

To date, seven coronaviruses are known to infect humans—HKU1, NL63, OC43, and 229E, which have been associated to mild respiratory and gastrointestinal symptoms, as well as SARS-CoV, MERS-CoV, and the novel emerging SARS-CoV-2, which share similar zoonotic features and are linked to severe disease outcomes, often in the context of epidemic and pandemic settings [6]. The latter three, aside from sharing their host-switching capacity and zoonotic origin, have also shown a propensity for increased pathogenicity. Such features are intimately related to complex evolutionary mechanisms, including high mutation rates and recombination signals that favor their adaptation process to different species [2,3]. After all diversity as seen in most emerging coronaviruses is a feature of host-switching viruses throughout their evolutionary paths. In the last two decades, the spillover of SARS-Coronavirus (SARS-CoV) in China (2002–2003), and MERS-Coronavirus (MERS-CoV) in the Middle East (2012–2016) have shown the deleterious effect of coronaviruses crossing the species barrier, their devastating effects on human health, and global impact following pandemic spreads [7,8].

Today, almost two decades after the emergence of SARS-CoV, the newly emerging Severe Acute Respiratory Syndrome Coronavirus 2 (SARS-CoV-2) is now spreading in pandemic proportions after the first reported case in the city of Wuhan, China on December 2019 [9]. This coronavirus-associated acute respiratory disease, the Coronavirus 2019 disease (COVID-19), has turned into a serious global health crisis with 6,407,129 infected individuals and 378,270 reported deaths by 2 June 2020. As the virus is vanishing from most industrialized countries in the northern hemisphere, a new spread wave is sweeping South America (SA) under more complex and heterogeneous epidemiological contexts that may favor increased transmission and long-term persistence [10]. In this sense, monitoring SARS-CoV-2 and an increased testing capacity becomes a critical issue for most South American nations.

The genome repertoire of SARS-CoV-2 is extensive (<30,000 nucleotides) as with most of the Coronaviruses that frequently recombine [11,12]. Its genome alignment is divided into two segment ORF, encoding both non-structural and four structural proteins arranged in the following order: spike (S), envelope (E), membrane (M), and nucleocapsid (N). Molecular detection of SARS-CoV-2 mainly relies on three regions with highly conserved sequences: (1) the *RdRp* gene (RNA-dependent RNA polymerase gene) in the open-reading frame ORF1ab region, (2) the *E* gene (envelope protein gene), and (3) the *N* gene (nucleocapsid protein gene), with both the *RdRP* and *E* genes exhibiting high analytical sensitivity for detection (technical limit of detection of 3.6 and 3.9 copies per reaction from in vitro transcribed RNA) [13,14], in contrast to the *N* gene, which exhibits much lower analytical sensitivity (8.3 copies per reaction) [14].

While thousands of whole-genome sequences are now available and easily accessible for close to real-time visualization in platforms such as NexStrain on the GISAID database (GISAID; https://www.epicov.org) (Elbe and Buckland-Merrett, 2017; Shu and McCauley, 2017), there has been a significant shortage on genomic information from SA ever since the virus first arrived in Brazil in March 2019. By 12 June 2020, entries from Brazil, Chile, Ecuador, Colombia, Uruguay, Peru, and Argentina have been deposited corresponding to 373 genomes available for this region [15]. However, there are no studies addressing genomic diversity and how this may impact molecular diagnosis of SARS-CoV-2. Thus, herein we compared the capacity of all currently accessible primer-probe sets to detect potential SARS-CoV-2 variants by analyzing against all available South American genomes while also assessing regional variation and how this may perhaps predict test performance by screening for potential mutations affecting regions harboring primer-probe binding sites.

## 2. Materials and Methods

The conservation of genomic targets of SARS-CoV-2 for molecular tests was evaluated in this study. As a first step, a revision about different schemes to detect viral infection by reverse real-time PCR was conducted, where we identified sets of primers and probes directed to the E, N, and RdRp genes (Table 1). In parallel, whole genome sequences of SARS-CoV-2 reported from South American countries were downloaded from the EpiCoVTM database of Global Initiative on Sharing All Influenza Data (GISAID) by 15 June 2020 [16], the most complete repository of genomic data of the pandemic coronavirus causing COVID-19. The complete set of 373 sequences was aligned using MAFFT v7.407 with an FFT-NS-2 algorithm and default parameter settings [17,18]. The sequence of severe acute respiratory syndrome coronavirus 2 isolate Wuhan-Hu-1 was included as a reference (access number NC_045512.2). The nucleotide diversity was determined at whole genome level from the alignment of selected genomes. A whole genome phylogenetic analysis was built in IQtree [2], considering IGT as the substitution model. The phylogenetic reconstruction was visualized in the interactive tool, Tree Of Life V4 (http://itol.embl.de). The multiple alignment obtained from whole genome sequences was used to extract sub-alignments for ach molecular target (N, E, and RdRp genes) in Unipro UGENE v.33.0 [19], considering the annotation of the isolate Wuhan-Hu-1 available in the National Center for Biotechnology Information (https://www.ncbi.nlm.nih.gov/nuccore/?term=Severe+acute+respiratory+syndrome+coronavirus+2+AND+Whuan). Each sub-alignment was manually verified to remove sequences with Ns, unidentified positions (denoted with the IUPAC code), and gaps. The "good sequence" alignment by genes (N, E, and RdRp) was used to identify nucleotide and haplotype diversity using DnaSP 5.10 software [20]. A representative sequence by haplotype was used to construct a haplotype alignment, and then trees were generated using FastTree double precision version 2.1.10 [21] and visualized in the interactive tool, Tree Of Life V4 (http://itol.embl.de) [22]. The genomic regions targeted for each scheme (primers+probe) were subsequently inspected, loading the alignment as metadata.

## 3. Results

A total of 16 oligonucleotide sets (primers+probes) were identified during the revision of schemes to detect SARS-CoV-2 infection using a molecular approach (Table 1). The parallel search of whole genome sequences in GISAID revealed a total of 747; however, most of these were duplicated. Verification of redundant sequences showed a total of 373 different genome sequences from the following seven South America countries: Argentina (n = 29), Brazil (n = 95), Colombia (n = 88), Ecuador (n = 4), Peru (n = 2), and Uruguay (n = 11). The results of the different diversity parameters evaluated are described in Table 2.

**Figure 1:**
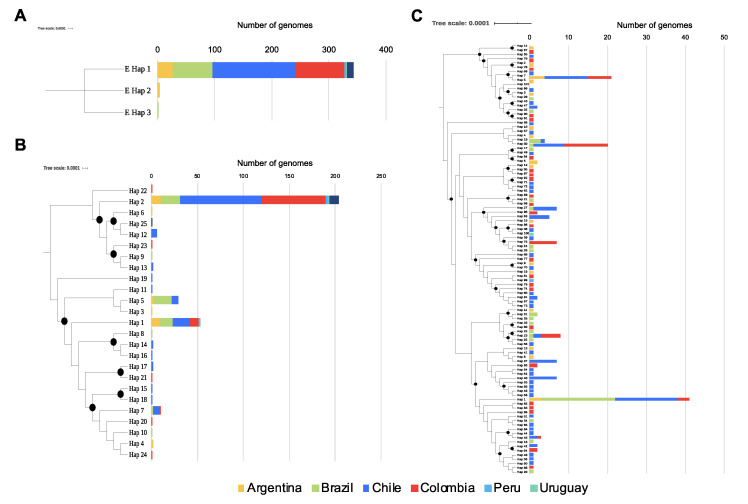
Distribution of haplotypes per gene in the different countries. (**A**). Gene E showed only three haplotypes, and haplotype 1 was the most frequent in the region (**B**). Gene N showed 25 different haplotypes, where haplotypes 1, 2, 5, and 7 were the most frequent in the region. (**C**). Gene RdRp showed 101 haplotypes, and was the most diverse gene used in the molecular diagnosis. The trees on the left of each panel show the relationships of the haplotypes, and the black dots indicate well-supported nodes (Bootstrap over 80%).

**Figure 2:**
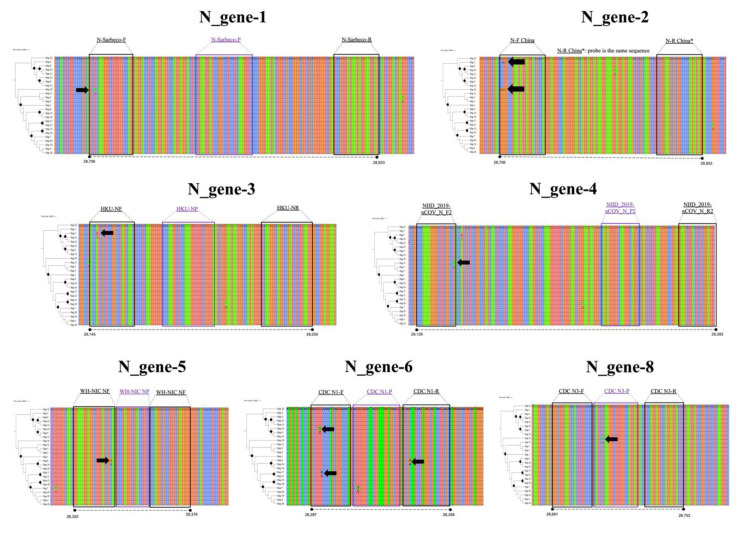
Multiple alignments of the N gene sequences. There were several polymorphisms identified in the annealing regions of the primers of three schemes (black arrows) (Corman, Jung, and CDC). The position in the reference sequence Wuhan-Hu-1 (NCBI Reference Sequence: NC_045512.2) are indicated below in each haplotype alignment. The trees on the left of each panel show the relationships of the haplotypes, and the black dots indicate well-supported nodes (Bootstrap over 80%).

**Figure 3:**
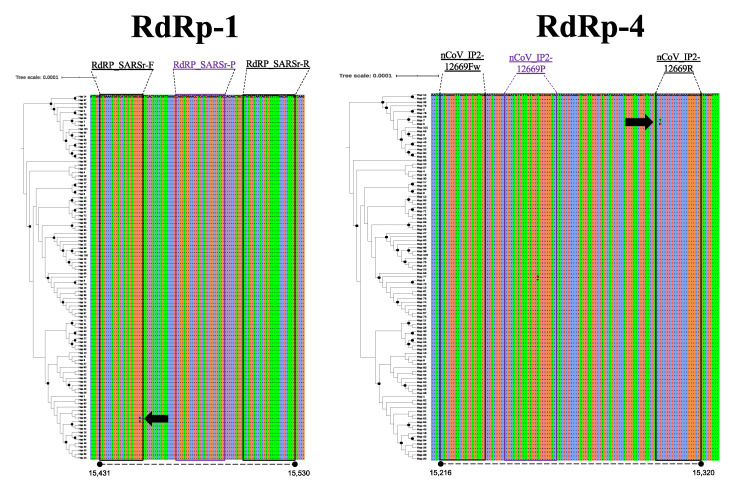
Multiple alignments of the RdRp gene sequences. There were several polymorphisms identified in the annealing regions of the primers of three schemes (black arrows) (Corman, Jung, and Pasteur). The position in the reference sequence Wuhan-Hu-1 (NCBI Reference Sequence: NC_045512.2) are indicated below in each haplotype alignment. The trees on the left of each panel show the relationships of the haplotypes, and the black dots indicate well-supported nodes (Bootstrap over 80%).

Concerning diversity, in the case of the E-gene, although three haplotypes were identified, only one of them (Hap-1) is predominant in all of the South American countries, with 99.13% of the total sequences (Figure 1A). No variable sites were found in the targets of the scheme flanking this genome region. In the case of the N-gene, a total of 25 haplotypes were identified (Figure 1B), with Hap-2, Hap-1, and Hap-5 being the most frequent (with 62.7%, 16.2%, and 8.9%, respectively). Variable sites were found in N_gene-6 to N_gene-8 schemes, this being the last part of the CDC-N scheme (primers+probe) and in the direct primers of the Jung schemes described in the different countries (China ‘N_gene-2’, Hong Kong ‘N_gene-3’, Japan ‘N_gene-4’, and Thailand ‘N_gene-6’) (Figure 2). Although most of these variable sites were detected in rare haplotypes, one variable site was detected in a dominant haplotype, involving two nucleotides in the Forward primer of the Jung-China scheme (N_gene-2) detected in Hap-2. In the case of ORF1ab (RdRp), a total of 101 haplotypes were detected in the analyzed dataset (Figure 1C), with Hap-1, Hap-7, and Hap-30 being predominant with 18.1%, 9.3%, and 8.8%, respectively. Only in the Forward primer of RdRp-1 were there detected variable sites for Hap-64 and Hap-65, and in the reverse primer of RdRp-4 for Hap-5 and Hap-7. Two areas with variable sites are marked with a black arrow in Figure 3.

**Figure 4:**
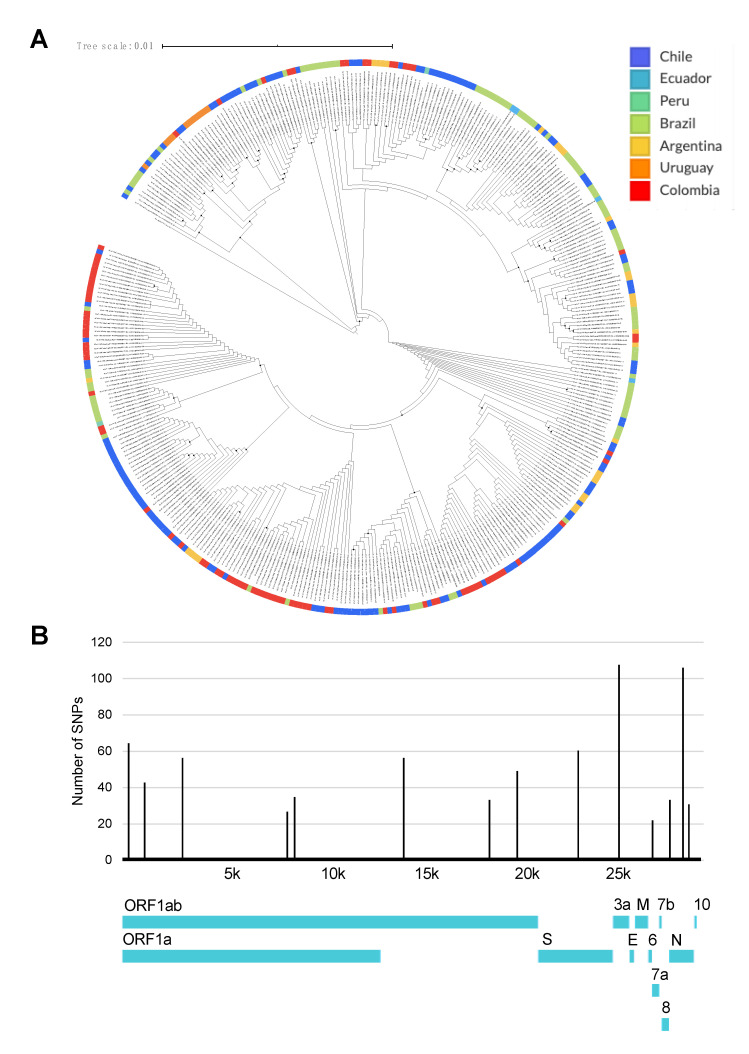
Relationships and nucleotide diversity of SARS-CoV2 genomes reported in South American countries. (**A**) Maximum likelihood tree of SARS-CoV2 genomes reported in GISAID database. The black dots represent well-supported nodes; (**B**) Nucleotide diversity per genome position determined from whole genome alignment of analyzed genomes. Whole genome sequence of SARS-CoV-2 Wuhan-Hu-1 strain (NCBI Reference Sequence: NC_045512.2) was included as a reference sequence for tree reconstruction, as well as nucleotide diversity analysis. This tree was constructed with the available genomes by 15 June, where to date (8 July 2020), around 687 new genomes are available.

We also analyzed the whole genomic diversity and SNPs frequency of SARS-CoV2 in SA, where the phylogenomic reconstruction showed that there was not a clustering by geography (Figure 4A) but that there is a significant number of SNPs in the ORF1a, S, 3a, 6, 8, and N genes (Figure 4B). Interestingly, the number of SNPs in the E gene was null.

## 4. Discussion

SARS-CoV2 has become one of the most important epidemics after the 1917–1918 “Spanish” influenza pandemic [27] with over 7 million infected individuals and close to 400,000 reported deaths worldwide [28]. To date, the majority of affected countries are developed nations with strong public health systems and up-to-date medical facilities which, despite this, have undergone severe hassles throughout the course of the pandemic. On the other hand, as the spread wave levels off in many regions of the world, other regions, such as South America, are confronting a significant rise in case numbers. Many developing countries have been affected by this novel coronavirus, particularly Brazil, Ecuador, Peru, and Colombia, which are clear examples that COVID-19 is taking a deadly toll [29].

These countries share many factors in common, such as marked poverty, lack of access to basic sanitation services, and inadequate healthcare facilities [10]. Such aspects are relevant in understanding the distinct shaping of the course with regard to epidemics and how it can differently affect the transmission dynamics of the virus. For example, the overcrowded environment of slums that make up most of the suburban areas in most South American cities preclude ideal social distancing efforts [10]. In addition, the inability to comply with quarantine measures due to stringent economic reasons, as well as the lack of water and appropriate sanitation policies, may favor transmission and halt potential mitigation efforts needed for containment of the virus [10]. This is why there is an urgent need to better understand and address the main drivers influencing the epidemic spread in such a heterogeneous scenario, alongside strengthening public health capabilities. Measures for assessing these scenarios include evaluating the available diagnostic tools for disease detection in order to improve outbreak response and contention.

In this study, we evaluated whether genomic diversity of SARS-CoV-2 could affect the performance of available primer-probe sets directed at the E, N, and RdRp genes, widely used nowadays for the molecular detection of SARS-CoV-2. Our results depict the ample genomic diversity present in the RdRp and N genes, which is a shared feature with other members of the coronaviridae family (Figure 1; Table 2; Figure 4B) [2,3]. In contrast, for most of the South American genomes, the E gene clustered within one haplotype with no SNPs present in these regions where different sets of primers and probes are known to anneal. This finding highlights the great utility of this gene as a screening target for diagnosis of SARS-CoV2 in the region. However, it is important to point out that these primer-probe sets may be susceptible to regional variations of the SARS-CoV2 virus.

A number of RT-PCR tests using different primer/probe sets targeting different regions of the viral genome have been and continue to be developed. The performance of these molecular tests is highly reliant on the primers, probes, and reagents used [23]. To date, sensitivities of these nucleic acid amplification tests (NAAT) are variable, and in many cases, less than optimal [30]. It has been proposed that genetic variations in the SARS-CoV-2 genome may play a role in the observed differences in sensitivity seen amongst different assays. Currently, there are no studies that evaluate the diagnostic performance of these NAATs in the region, and future studies should consider focusing on understanding their diagnostic operational capabilities to be massively used to detect SARS-CoV2 in SA.

Current available data, at a global scale, reveal that SARS-CoV-2 has accumulated moderate genetic diversity [31]. As opposed to other RNA viruses, COVs exhibit a more modest mutation rate, primarily because of their proofreading capacity and greater replication fidelity [32]. Considering the reported underlying global diversity of SARS-CoV-2 to date, its mutation rate has been estimated in 2 of ~6 × 10^4^ nucleotides/genome/year [30] with the majority of these mutations described so far as neutral mutations [33]. Regional variations in SARS-CoV-2 diversity has been recognized as a dynamic process of functional and ongoing adaptation of the virus to the human host [31], as well as a result of multiple and variable sources of introduction to other regions [15]. Such is the case of South America, where recent phylogenetic analyses suggest that most viruses have entered from Europe, Oceania, and in a less proportion from Asia [15]. Even though sequence variation among SARS-CoV-2 isolates remains moderate, several consistent mutation hot spots have been identified in specific locations that are critical target regions for viral detection (Figure 4). Herein, we highlight several mutations in the ORF1 and N genes that are widely used to detect the virus in the region representing a plausible effect on the false-negative rate.

A comparative analysis study by Jung et al. [23] showed that a combination of ORF1 ab (China), N2, N3 (USA), and NIID (Japan) displayed the most sensitive and reliable amalgamation of detection targets [23]. Similarly, Wang et al. also reported the occurrence of mutations in the ORF1a (nt8782), ORF8 (nt28144), and N (nt29095) regions, contrasting with the E, 6, and 7b regions which exhibited a solid conservation with no mutations detected [23]. Comparable findings by Nalla et al. comparing seven different primer-probe sets also showed the most sensitive assays that targeted the E and N2 genes [25]. This supports our findings on the right selection of the E gene for a massive screening of SARS-CoV2 in SA, due to its low genetic variation (Table 1; Figure 2; Figure 4).

Previous studies from South America have also identified changes in the ORF1ab, E, and nucleocapsid genes, occurring at the level of specific oligo-binding sites which could negatively impact adequate viral identification. Despite the relative conservation of the E-gene [30], as also shown by our study, variants associated with a C-to-T transition at position 26,340 have shown to affect testing performance in commercial assays, such as the cobas^®^ SARS-CoV-2 (Roche) [34]. The fact that this mutation has emerged independently on various occasions adds a word of caution when relying uniquely on this target region, given the latent possibility of mutations that could potentially limit probe binding and impair amplification [34].

As a matter of fact, the Pan-American Health Organization (PAHO), in their most recent guidelines for the detection and diagnosis of COVID-19 virus infection, stated that although a dual-target testing approach using different genetic markers (E, N, or RdRp genes) is recommended, once local circulation is confirmed and widespread though a region, single target testing may be implemented as long as curves and other quality assurance specifications are met appropriately [35]. Amongst all current proposed targets, the PAHO recommends the use of E or RdRP genes for diagnosis, prioritizing the E gene for single-target testing, given its slight higher sensitivity [35]. In light of this recommendation and given the absence of reported SNPs within the E gene from over 300 South American genomes analyzed so far, the E gene stands as the most promising candidate and first-line screening tool for the molecular diagnosis of SARS-CoV2 in the region.

Regarding results for the N and RdRp genes, several haplotypes were identified in the analyzed genomes (Figure 1; Table 2). Of interest, we identified that diversity across all South American SARS-CoV2 sequences apportioned in at least three different haplotypes for these two genes, portraying the heterogeneity of the circulating strains throughout the region [16] and the potential role of these proteins in viral pathogenesis [15,36,37]. When screening for SNPs across different primer-probe binding regions, we identified several SNPs capable of altering physicochemical properties of the PCR assays (Figure 2 and Figure 3). This diversity, as well as its ability to negatively influence diagnostic test performance, is a shared feature with other coronaviruses [30,38]. As mentioned earlier, this is the reason why the occurrence of false-negative results in the context of ongoing epidemics should be taken cautiously. Evidence from Europe on the occurrence of false-negative results for N-gene-based assays support our in silico findings [39,40]. Despite no present evidence on false-negative results for RdRp gene-based assays, its inherent mutation rate (due to environmental pressure and its role as a virulence factor), and considering how this gene showed low sensitivity [41] is an aspect that deserves further investigation.

It is important to note some limitations in the interpretations of our data. First, because all sequences were retrieved from public databases, the accuracy of these sequences could not be entirely verified. In addition, given the most recent emergence of the virus in South America, it is possible that our analysis may represent a snapshot of the most recent evolutionary episodes and not the entire developmental history of the different lineages since their introduction. Further studies will be needed to expand our ability to characterize the complete and evolving evolutionary track of the virus.

In summary, this preliminary analysis based on the genomic diversity of SARS-CoV-2 in South America demonstrates how the presence of changes in suggested target regions for primer annealing sites may preclude accurate molecular diagnosis of SARS-CoV-2 when targeting locations within the N-gene region. Our results confirm the relatively conserved fitness of the E-gene region where no mutations were found, thus making it an ideal candidate for first-line screening in South America. Future studies should consider a specific primer and probe design over these genes that encompass the known diversity of SARS-CoV2 in South America. Due to the lack of resources and unavailability to acquire reagents and consumables for molecular diagnosis in many areas of South America, the implementation of a single marker assay proves a feasible and cost-effective option for diagnostics in resource-depleted countries. Future studies should unveil the diagnostic performance in situ of the E gene across multiple geographical regions.

## Figures and Tables

**Table 1:** Sequences of primers and probes directed to E, N, and RdRp genes and used for the molecular detection of SARS-CoV-2.

ID		Name	Sequences (5’-3’)	Reference
	1	RdRP_SARSr-F	GTGARATGGTCATGTGTGGCGG	[13]
RdRp		RdRP_SARSr-R	CARATGTTAAASACACTATTAGCATA	
		RdRP_SARSr-P	FAM- CAGGTGGAACCTCATCAGGAGATGC-BHQ1	
	2	ORF1ab-F (China)	CCCTGTGGGTTTTACACTTAA	[23]
		ORF1ab-R (China)	ACGATTGTGCATCAGCTGA	
		ORF1ab-Probe (China)	CCGTCTGCGGTATGTGGAAAGGTTATGG	
	3	HKU-ORF1b-nsp14F (Hong Kong)	TGGGGYTTTACRGGTAACCT	
		HKU-ORF1b-nsp14R Hong Kong	AACRCGCTTAACAAAGCACTC	
		HKU-ORF1b-nsp14P Hong Kong	TAGTTGTGATGCWATCATGACTAG	
	4	nCoV_IP2-12669Fw	ATGAGCTTAGTCCTGTTG	[24]
		nCoV_IP2-12759Rv	CTCCCTTTGTTGTGTTGT	
		nCoV_IP2-12696 Probe (+)	AGATGTCTTGTGCTGCCGGTA [5’]Hex [3’]BHQ-1	
	5	nCoV_IP4-14059Fw	GGTAACTGGTATGATTTCG	
		nCoV_IP4-14146Rv	CTGGTCAAGGTTAATATAGG	
		nCoV_IP4-14084 Probe (+)	TCATACAAACCACGCCAGG [5’]Fam [3’]BHQ-1	
		nCoV_2019 Forward	CAAATTCTATGGTGGTTGGCACA	[25]
	6	nCoV_2019 Reverse	GGCATGGCTCTATCACATTTAGG	
		nCoV_2019 Probe	FAM- ATAATCCCAACCCATRAG-MGB	
N-gene	1	N_Sarbeco_F	CACATTGGCACCCGCAATC	[13]
		N_Sarbeco_R	GAGGAACGAGAAGAGGCTTG	
		N_Sarbeco_P	FAM- ACTTCCTCAAGGAACAACATTGCCA-BHQ1	
	2	N-F (China)	GGGGAACTTCTCCTGCTAGAAT	[23]
		N-R (China)	CAGACATTTTGCTCTCAAGCTG	
		N-probe (China)	CAGACATTTTGCTCTCAAGCTG	
	3	HKU-NF (Hong Kong)	TAATCAGACAAGGAACTGATTA	
		HKU-NR (Hong Kong)	CGAAGGTGTGACTTCCATG	
		HKU-NP (Hong Kong)	GCAAATTGTGCAATTTGCGG	
	4	NIID_2019-nCOV_N_F2 (Japan)	AAATTTTGGGGACCAGGAAC	
		NIID_2019-nCOV_N_R2 (Japan)	TGGCAGCTGTGTAGGTCAAC	
		NIID_2019-nCOV_N_P2 (Japan)	ATGTCGCGCATTGGCATGGA	
	5	WH-NIC N-F Thailand	CGTTTGGTGGACCCTCAGAT	
		WH-NIC N-R Thailand	CCCCACTGCGTTCTCCATT	
		WH-NIC N-P Thailand	CAACTGGCAGTAACCA	
	6	CDC N1 Forward	GACCCCAAAATCAGCGAAAT	[26]
		CDC N1 Reverse	TCTGGTTACTGCCAGTTGAATCTG	
		CDC N1 Probe	FAM- ACCCCGCATTACGTTTGGTGGACC-BHQ1	
	7	CDC N2 Forward	TTACAAACATTGGCCGCAAA	
		CDC N2 Reverse	GCGCGACATTCCGAAGAA	
		CDC N2 Probe	FAM- ACAATTTGCCCCCAGCGCTTCAG-BHQ1	
	8	CDC N3 Forward	GGGAGCCTTGAATACACCAAAA	
		CDC N3 Reverse	TGTAGCACGATTGCAGCATTG	
		CDC N3 Probe	FAM- AYCACATTGGCACCCGCAATCCTG-BHQ1	
E-gene		E_Sarbeco_Forward	ACAGGTACGTTAATAGTTAATAGCGT	[13]
		E_Sarbeco_Reverse	ATATTGCAGCAGTACGCACACA	
		E_Sarbeco_Probe	FAM- ACACTAGCCATCCTTACTGCGCTTCG-BHQ1	

**Table 2:** Nucleotide and haplotype diversity of genomic regions of SARS-CoV-2 used for molecular detection.

Parameter	E-Gene	N-Gene	RdRp (ORF1ab Polyprotein Region)
Number of sequences	348	327	228
Number of sites	228	1260	21332
Number of variable sites	2	25	131
Number of haplotypes (h)	3	25	101
Haplotype diversity (Hd)	0.023	0.573	0.9475
Nucleotide diversity (Pi)	0.00010	0.00135	0.00015
Theta (per site) from Eta	0.00136	0.00312	0.00102
